# Measuring CMAPs in addition to MEPs can distinguish peripheral ischemia from spinal cord ischemia during endovascular aortic repair

**DOI:** 10.1016/j.cnp.2020.11.002

**Published:** 2020-12-11

**Authors:** Fabian I. Kerkhof, Jan van Schaik, Richard A. Massaad, Catharina S.P. van Rijswijk, Martijn R. Tannemaat

**Affiliations:** a Department of Neurology, Leiden University Medical Center, the Netherlands; b Department of Surgery, Leiden University Medical Center, the Netherlands; c Department of Interventional Radiology, Leiden University Medical Center, the Netherlands

**Keywords:** Neuromonitoring, Endovascular thoracoabdominal aneurysm repair, Motor evoked potential, Compound muscle action potential, Spinal cord ischemia

## Abstract

**Objective:**

Spinal cord injury is a devastating complication after endovascular thoracic and thoracoabdominal aneurysm repair (EVAR). Motor evoked potentials (MEPs) can be monitored to detect spinal cord injury, but may also be affected by peripheral ischemia caused by femoral artery sheaths. We aimed to determine the incidence of peripheral ischemia during EVAR, and whether central and peripheral ischemia can be distinguished using compound muscle action potentials (CMAPs).

**Methods:**

We retrospectively analyzed all EVAR procedures between March 1st 2015 and January 1st 2020 during which MEPs were monitored. Peripheral ischemia was defined as both a reduction in MEP amplitudes reversed by removing the femoral sheaths and no clinical signs of immediate post-procedural paraparesis. All other MEP decreases were defined as central ischemia.

**Results:**

A significant MEP decrease occurred in 14/27 (52%) of all procedures. Simultaneous CMAP amplitude reduction was observed in 7/8 (88%) of procedures where peripheral ischemia occurred, and never in procedures with central ischemia.

**Conclusions:**

MEP reductions due to peripheral ischemia are common during EVAR. A MEP-reduction without a CMAP decrease indicates central ischemia.

**Significance:**

CMAP measurements can help to distinguish central from peripheral ischemia, potentially reducing the chance of misinterpreting of MEP amplitude declines as centrally mediated, without affecting sensitivity.

## Highlights

• MEP amplitude decreases occur frequently during endovascular aortic aneurysm repair.
• They are usually caused by peripheral ischemia due to femoral artery sheaths.
• Peripheral and central ischemia can be distinguished by measuring CMAP amplitudes.

## Abbreviations

EVAR: endovascular repair of thoracic or thoracoabdominal aortic aneurysm
IONM: intra-operative neuromonitoring
MEP: motor evoked potential
SCI: spinal cord ischemia
TAA: thoracoabdominal aortic aneurysm
TOF: train of four
CMAP: compound muscle action potential

## 1 Introduction

Spinal cord injury (SCI) is a devastating complication after thoracic or thoracoabdominal aneurysm (TAA) repair. Although endovascular repair appears to carry a lower risk of SCI than open surgery, reported rates of permanent SCI after endovascular thoracic and thoracoabdominal aneurysm repair (EVAR) still range between 2 and 11% (Miranda et al., 2018).

Intra-operative neuromonitoring (IONM) is increasingly used to detect intra-operative ischemic events involving the spinal cord. Motor evoked potentials (MEPs) are generated by transcranial stimulation of the corticospinal tract (or upper motor neuron), resulting in a descending volley synapsing on the alpha motor neuron in the ventral horn of the spinal cord (or lower motor neuron), which in turn activates the muscles from which MEPs are recorded (Macdonald, 2006). This technique can be used to detect ischemia of the spinal cord before irreversible damage occurs and is therefore commonly used during aortic repair surgery (Greiner et al., 2012, Hattori et al., 2019, Jacobs et al., 2006). During EVAR, MEPs can also be used to guide treatment options available such as correcting hemoglobin levels >7 mmol/L, increasing blood pressure, drainage of cerebrospinal fluid or staging the procedure (Malloy et al., 2020, Schurink et al., 2013).

During EVAR, a reduction in MEP amplitudes can also be caused by peripheral ischemia of the leg, due to the large size of the sheaths used to gain vascular access, leading to temporary occlusion of the femoral artery, causing ischemia of the leg. Under local ischemia, MEPs in the occluded extremity disappear (Husain, 2008). Spinal cord ischemia can have an asymmetric onset, making it difficult to distinguish between central (spinal cord) and peripheral (leg) ischaemia (Mehmedagic et al., 2013). Incorrect interpretation of a decrease in MEP amplitudes may lead to unnecessary reactions, such as preventive measures or even termination of the procedure. Normalisation of MEP amplitudes after removing the sheath can provide definite proof that the observed reduction in MEP amplitudes was caused by peripheral ischemia, but this is not an option during the procedure.

Train of four (TOF) testing is used in anaesthesia to aid the dosing of neuromuscular blockers. During IONM, a TOF is commonly used early in the procedure to determine whether MEP amplitudes are still affected by neuromuscular blockers. A TOF consists of four supramaximal electrical stimuli to a peripheral nerve, generating four compound muscle action potentials (CMAPs). A decrement in subsequent CMAP-amplitudes then indicates an impairment of neuromuscular transmission (McCoy et al., 1995). We hypothesized that, since both the stimulus and recording site used to generate CMAPs only involve peripheral axons, and SCI only affects the relevant spinal cord motor neurons and descending corticospinal tracts, CMAP amplitudes should be unaffected by SCI. In contrast, under local ischemia a peripherally generated CMAP will decrease in amplitude, and eventually disappear altogether, as has been demonstrated in rats (Schmelzer et al., 1989).

In this retrospective study, we aimed to determine the incidence of peripheral ischemia during EVAR of thoracic and thoracoabdominal aortic aneurysms, and whether the amplitude of the CMAP of distal leg muscles can be used to help distinguish between central and peripheral ischemia.

## 2 Methods

### 2.1 Study design

Retrospective study on a consecutive patient series. Dutch law does not require individual informed consent or formal evaluation by an ethics committee for scientific research on anonymous data gathered for the purpose of clinical patient care.

### 2.2 Patients

All patients treated with EVAR for thoracic or thoracoabdominal aneurysm in the Leiden University Medical Centre (LUMC) between March 1st 2015 and December 31st 2019 who underwent IONM with measurements of MEPs during the procedure were included. To determine the incidence of MEP reduction each procedure was included once, regardless of whether a MEP reduction occurred in one leg or both. We recorded age and sex of all patients. A subgroup of patients, for whom CMAP measurements were available both at the start of the procedure and at the time of significant MEP amplitude decreases, was used to determine the diagnostic yield of CMAP measurements for the identification of peripheral ischemia. In these patients, CMAP-amplitude measurements were performed intermittently, at the discretion of the attending technician based on an ad hoc assessment of their relevance. For this analysis, MEP reductions in each limb were analysed as separate events.

Prior to the procedure, all patients were evaluated by a neurology resident or physician assistant for assessment of motor and sensory function. All patients were assessed by a vascular surgeon after recovery from anaesthesia. In case of a clinical suspicion of spinal cord injury, they were evaluated by a neurology resident within 24 h after the procedure.

### 2.3 Monitoring procedures

During all procedures, MEPs were recorded intraoperatively, using a NIM Eclipse IONM system (Nim Eclipse 4.2, Medtronic, Minneapolis) and Medtronic needle electrodes. MEPs were elicited using a fast TCeMEP train, with a train count of five and a train rate of 333 stimuli/second, and recorded from several leg muscles on both sides. Selection of recording sites differed between operations, but in the included procedures always included the flexor/abductor hallucis, vastus medialis and tibialis anterior muscles, and the gastrocnemius muscle in nine out of ten cases. To distinguish a diffuse significant MEP-amplitude decrease from a significant MEP-amplitude decrease in the legs only, intrinsic hand muscles were monitored in all procedures. Patients were anesthetized using propofol. To facilitate intubation, patients were given a one-time bolus of 25 to 30 mg of rocuronium. CMAPs were measured at the start of the procedure in the context of a TOF measurement, which was performed in order to determine whether any appreciable level of neuromuscular blockade remained. When no decremental response or a decrement no greater than 5% was found this was taken as evidence that this was not the case. When a decrement larger than 5% remained rocuronium was antagonized using sugammadex. When deemed relevant by the attending technician, CMAPs were determined at the time of significant decrease in MEP amplitudes. CMAPs were evoked by supramaximal stimulation of the tibial nerve at the ankle, and recorded from the flexor/abductor hallucis muscle(s). In order to ensure supramaximal stimulation of the tibial nerve, stimulus intensity was increased until there was no further increase in CMAP-amplitude, after which a small increase (5–10 mA) in intensity was added to ensure supramaximal stimulation. After this no alterations were made to stimulus intensity. The approximate timing of introduction and removal of the large femoral sheaths was not systematically recorded during the procedure. When femoral sheath removal was recorded, this information was depicted graphically and noted in the results section.

### 2.4 Definitions

A significant reduction in MEP amplitudes was defined as a decrease in the peak-to-peak amplitude of at least 50% relative to a previously established baseline in a combination of three leg muscles, not explained by other obvious factors, e.g. the use of inhalation anaesthetics (Husain, 2008).

The CMAP amplitude was defined as the amplitude of the first CMAP elicited in the context of a TOF measurement. Analogous to the definition of a MEP-amplitude reduction, a reduction in CMAP-amplitude of at least 50% was taken as significant.

Peripheral ischemia was defined as both a reduction in MEP amplitudes reversed by removing the femoral sheaths and no immediate clinical signs of post-procedural paraparesis. All other cases of MEP amplitude reduction were defined as central ischemia. Delayed paralysis/paresis was defined as paralysis or paresis occurring after the patient had initially shown good motor scores postoperatively.

### 2.5 Analysis

Data were exported to Excel and re-analyzed using a custom made script in R Core Team version 2019. All amplitudes were calculated peak to peak. To facilitate comparison of changes in MEP-amplitude to those in CMAP-amplitudes, amplitudes were normalized to a baseline, using the average of the first two maximal amplitudes from the sequence used for determining the stimulus intensity needed for MEPs at the start of the procedure. Percentages relative to baseline were averaged for the MEP amplitudes of the flexor/abductor hallucis, gastrocnemius and tibialis anterior muscles. For CMAP-amplitudes the first two non-decrementing responses were used for baseline values. Amplitude changes were graphically displayed using GraphPad Prism 8.

### 2.6 Statistics

For Table 1, p-values of differences in age, gender and BMI between groups were determined using Pearson’s chi-squared test, for the ASA score and Crawford classifications a multiple factor ANOVA was used. Calculations were made using IBM SPSS statistics version 24.

**Table 1:** Patient characteristics of all included patients. Two patients underwent an endovascular thoracoabdominal aneurysm repair twice, one of these procedures was included in the group of patients with CMAP measurements.

		All patients (n = 18)	Patients with CMAP measurements (n = 7)	P-value
Age (years), median		72.9	72.1	0.814
Female		5 (28%)	4 (57%)	0.170
BMI (kg/m^2^), median		26.4	25.7	0.759
ASA score	I-II	3	1	0.884
	III-IV	15	6	
	V-VI	0	0	
Crawford classification	I	5	0	0.130
	II	6	4	
	III	3	3	
	IV	4	0	

## 3 Results

### 3.1 Incidence of peripheral and central ischemia

**Fig. 1:**
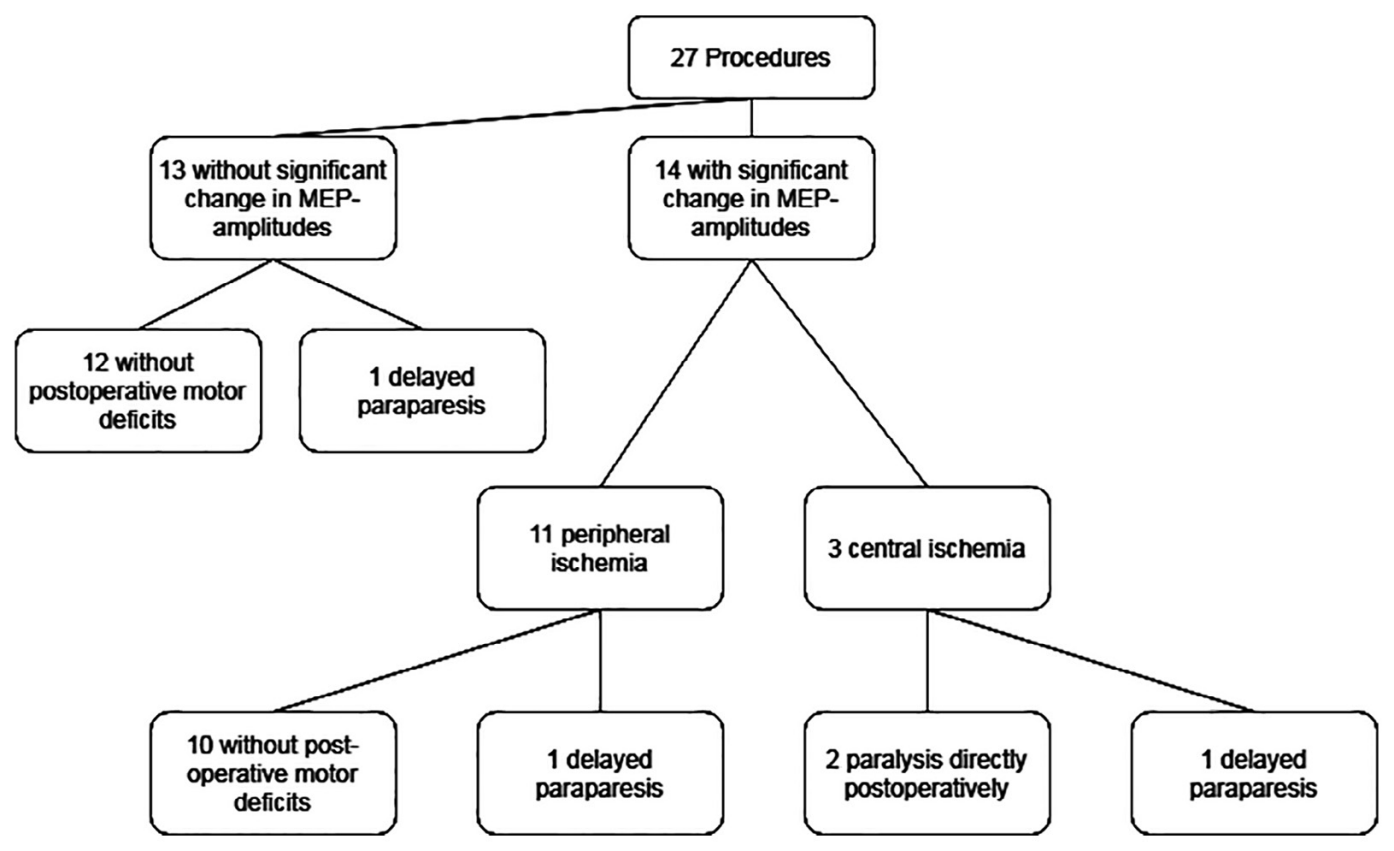
Flowchart of included procedures.

During the study period, 27 EVAR procedures with IONM took place, involving 25 patients. In five patients a thoracic repair, and in twenty patients a thoracoabdominal repair was performed. Baseline characteristics are presented in Table 1. There were no significant differences between the group of seven patients with CMAP measurements and eighteen patients without CMAP measurements. In thirteen procedures, no significant MEP amplitude reduction occurred. None of these patients had a postoperative paralysis or paresis, although one of them developed a paresis later, at an unknown time within four days after the procedure. This was classified as delayed paresis. In fourteen procedures (52%), a significant MEP amplitude reduction occurred. In eleven of these (79%), the amplitude reductions were deemed to be caused by peripheral ischemia: MEPs returned to baseline amplitudes after removal of the femoral sheaths, and patients did not have any direct post-procedural neurological deficits. One of these eleven patients did develop a paresis of both legs of unknown cause but presumed to be of an embolic origin twelve hours after surgery, which was classified as delayed paresis. In three procedures MEP-amplitude decline was caused by central ischemia. MEPs did not recover after the removal of the sheaths. Two of these patients demonstrated paralysis of both legs directly postoperatively; one did not have any immediate neurological deficits, but developed a paraparesis 10 days after surgery (see Fig. 1).

### 3.2 MEP and CMAP changes

Out of 39 baseline MEP amplitudes, 36 were >200 µV. Three amplitudes were <200 µV, all from vastus medialis muscles. Mean (±standard deviation) MEP amplitudes were: 3189 ± 2886 µV for the flexor hallucis muscle, 743 ± 324 µV for the gastrocnemius muscle, 1709 ± 707 µV for the tibialis anterior muscle, and 345 ± 202 µV for the vastus medialis muscle. Baseline mean CMAP amplitudes were all >2500 µV, with a mean of 5855 ± 3691 µV.

In a subset of seven procedures (ten legs, as CMAPs were measured bilaterally in three patients, all with peripheral ischemia), we obtained CMAP measurements during the period of MEP amplitude reduction. Eight of these reductions were deemed to be caused by peripheral ischemia (patients 1–5) and two were caused by central ischemia (patients 6 and 7). MEP amplitudes declined bilaterally in both patients with central ischemia: in patient 6 the amplitude decline occurred simultaneously, in patient 7 there was a delay between the right and the left leg, with the amplitude decline in the right leg occurring 60 min before the decline in the left leg. A substantial, simultaneous reduction of CMAP amplitudes was observed in seven legs with peripheral ischemia and none of the patients with central ischemia.

**Fig. 2:**
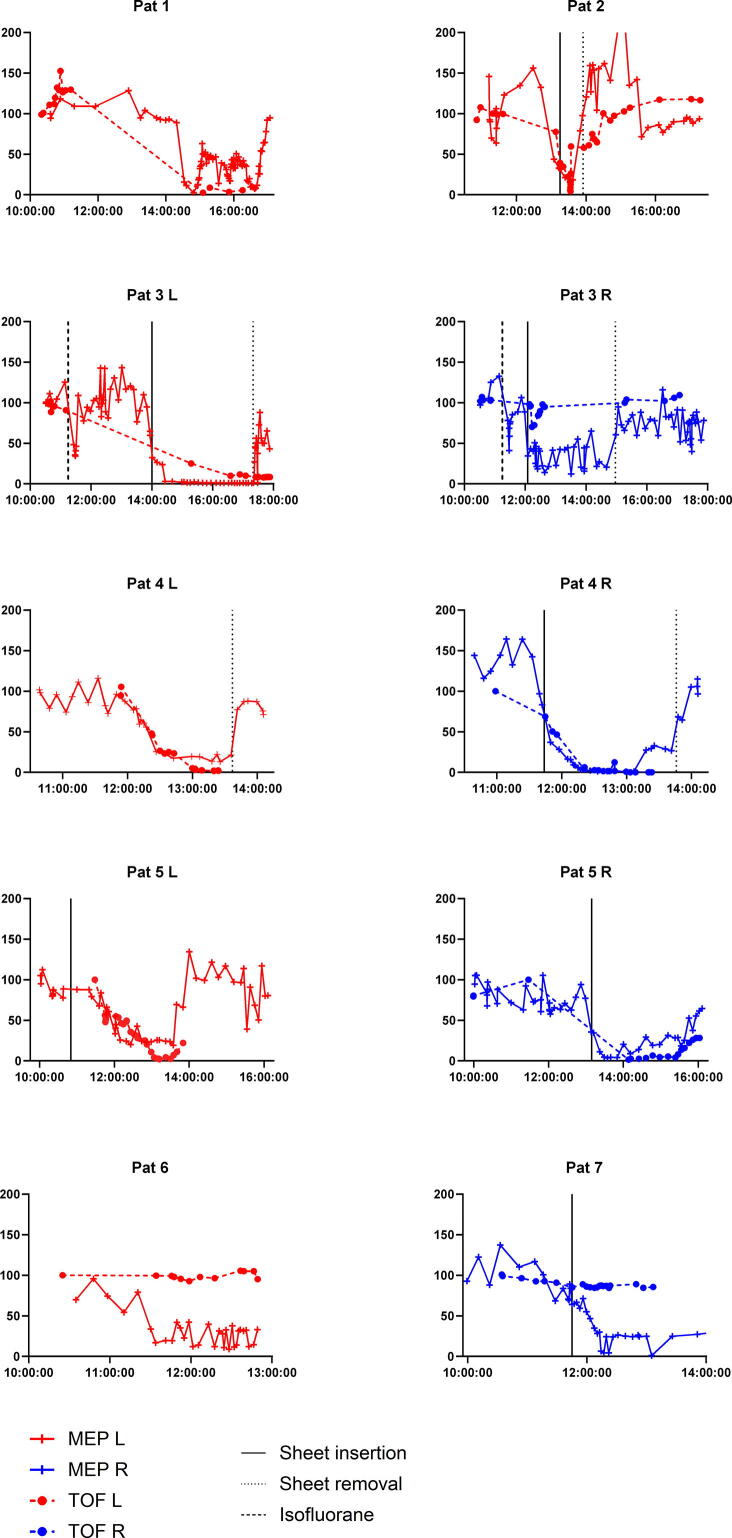
Relative amplitude changes of MEPs and CMAPs for seven patients. “MEP L” and “MEP R” are averaged percentages of the tibialis anterior, gastrocnemius, vastus medialis and flexor/abductor hallucis muscles, “CMAP L” and “CMAP R” the amplitudes of the left and right CMAP. X-axis shows the time of day. Times of sheath insertions and removals are indicated by vertical lines. Data on sheet removal and insertion were not always available, and indicated times are sometimes based on estimates. Patients 1–5 were classified as peripheral ischemia, patients 6 and 7 had central ischemia. Note that in patient 3, the CMAP amplitude did not decrease on the right side, although the MEP reduction was classified as peripheral ischemia (the MEP returned after removal of the sheath and the patient had no paresis).

MEP and CMAP amplitude decreases as well as recoveries appeared to occur simultaneously in 6 measurements in patients 1, 2, 4 and 5 (Fig. 2). In one patient (patient 3), the administration of isoflurane caused a brief reduction of MEP amplitudes, which recovered completely after several minutes. Information on insertion of sheaths was available in 5 out of 7 patients (7 out of 10 measurements), information on removal of sheaths was available in 3 out of 7 patients (5 out of 10 measurements). It is likely that information on the exact timing of the insertion of sheaths was not exact but rather delayed, since information on insertion of sheaths is often only gathered by the technician when prompted to do so, i.e. when a decline in MEP amplitude was observed. Both MEP and CMAP amplitudes recovered equally fast after removal of the sheaths in 3 patients. In patient 3, results appeared to be mixed: whereas the MEPs and CMAPs on the left side show a simultaneous amplitude reduction, the MEPs of the right leg decrease in amplitude more severely and for a longer duration than the CMAPs on the right side. In patients 6 and 7, who suffered from spinal cord ischemia, significant MEP amplitude decreases occurred while distally generated CMAP amplitudes were essentially unchanged. Fig. 2 presents the amplitude changes over the course of the procedure for all procedures.

## 4 Discussion

In this retrospective study we show that a significant decrease in MEP amplitudes is a very common event, and usually occurs asymmetrically. In the majority of procedures these decreases are caused by peripheral ischemia. The distinction between central and peripheral ischemia is important, as peripheral ischemia is reversible, whereas central ischemia may lead to permanent paraplegia. In addition, central ischemia is likely to necessitate immediate action during the procedure to reduce the risk of permanent paresis. Here, we deliberately applied a very narrow definition of peripheral ischemia (both normalization of MEP amplitudes after removal of the femoral sheaths and clinical absence of immediate post-procedural paresis) to reduce the chance that cases of central ischemia would be misclassified as peripheral ischemia.

Delayed ischemia occurred in three patients, both with and without per-procedural MEP changes, showing that delayed ischemia is a complication that is particularly difficult to detect or prevent with MEP monitoring. This is likely due to the fact that the occurrence of spinal cord ischemia is a dynamic and complex process, with some factors only arising after the procedure, while MEPs give an indication of current spinal tract function only. We therefore classified patients based on immediate post-procedural neurologic function as this is likely to provide the best correlate of per-procedural monitoring results. Nonetheless, per-procedural signs of central ischemia probably indicate an increased risk for developing delayed paresis, even when they are temporary (Acher et al., 2008, Guerit and Dion, 2002).

Our data show that CMAP measurements, which are usually already monitored as part of TOF measurements, seem to be useful for distinguishing peripheral from central ischemia. Not all patients with peripheral ischemia were identified correctly, as CMAP amplitudes appeared to remain unchanged in one leg with peripheral ischemia (patient 3, right leg). However, it should be noted that the number of CMAP measurements in this patient was low, and a temporary decrease in CMAP amplitude might have been observed if measurements were performed more frequently during the period of reduced MEP amplitudes. Importantly, a combined MEP/CMAP intraprocedural control did not affect sensitivity (i.e. no cases of spinal cord ischemia were missed compared to MEP measurements alone) and therefore appears to be a very useful test during EVAR. Even though the quality and quantity of our data are insufficient to warrant immediate application of this technique, these promising first results justify further prospective studies on this subject. Furthermore, the measurement of CMAPs is not harmful to the patient, does not require changes in the monitoring setup and can be performed with little effort and no extra costs.

### 4.1 Limitations

Due to the retrospective nature of this study, we were limited to the use of data recorded during routine clinical practice of monitoring in an operating room. As a result, CMAP amplitudes were not determined in all procedures with MEP reductions and they were not monitored at regular intervals. This limited the total number of inclusions, which affects the generalizability of the results. Furthermore, sheath insertions and removals were not always noted, or the information did not contain the exact time of insertion or removal. Generally, when MEPs returned, CMAP measurements were no longer performed, so this study does not allow conclusions on the temporal relation between MEP and CMAP after recovery from peripheral ischemia.

In addition, the gold standards used in this study for peripheral and central ischemia may not have been perfect: patients in whom MEP amplitudes recovered shortly after sheath removal and with no signs of paresis immediately postoperatively were classified as having (temporary) peripheral ischemia. However, central ischemia can also be temporary, for instance due to a combination of suboptimal perfusion of the spinal cord and low blood pressure, and in these cases patients may have been misclassified as having peripheral ischemia when the recovery of MEP amplitudes coincided by chance with removal of sheaths. Furthermore, temporary central ischemia may be masked by concomitant peripheral ischemia and central ischemic events cannot be monitored while MEPs are absent due to peripheral ischemia. However, none of the patients in the “peripheral ischemia” group showed any signs of post-procedural paresis immediately after surgery. This suggests that our classification is useful in clinical practice, although it should be noted that the presence of MEPs at the end of the procedure does not rule out the possibility of delayed paresis, which may occur as a result of embolic events or hypotensive episodes after the procedure.

### 4.2 Future research perspectives

In order to further establish distal CMAP measurements as a valid method of distinguishing between peripheral and central ischemia further research should come in the form of a larger, prospective study. Additional information could be produced by keeping to a more structured measurement regimen and record keeping specifically aimed at the relation between MEPs and CMAPs, and the timing of any changes relative to events that can cause peripheral and/or central ischemia. Information on the exact timing and depth of peripheral ischemia could be gained from measuring femoral blood flow and oxygen saturation levels in the leg.

## 5 Conclusion

During endovascular thoracic and thoracoabdominal aortic aneurysm repair, MEP amplitude reductions occur frequently, usually asymmetrically, as the result of peripheral ischemia during femoral sheath placement. Despite the small number of subjects our data show that CMAP measurements seem to be useful for distinguishing peripheral from central ischemia: by incorporating a CMAP measurement, only three out of ten MEP-reductions were identified as potentially of central origin, without affecting sensitivity. Measuring CMAPs does not require changes in the monitoring setup and can be performed with little effort and no extra costs.

## 6 Data statement

Raw data will be made available upon request, within the limits of applicable laws concerning patient privacy.

## Declaration of Competing Interest

The authors declare that they have no known competing financial interests or personal relationships that could have appeared to influence the work reported in this paper.
